# Reconstruction of Purine Metabolism in Bacillus subtilis to Obtain the Strain Producer of AICAR: A New Drug with a Wide Range of Therapeutic Applications 

**Published:** 2011

**Authors:** K.V. Lobanov, L. Errais Lopes, N.V. Korol’kova, B.V. Tyaglov, A.V. Glazunov, R.S. Shakulov, A.S. Mironov

**Affiliations:** State Research Institute for Genetics and Selection of Industrial Microorganisms

**Keywords:** anticancer agent AICAR, purine metabolism, genome reconstruction, *Bacillus subtilis *strain - producer of AICAR

## Abstract

AICAR is a natural compound, an analogue and precursor of adenosine. As activator of AMP-activated protein kinase (AMPK), AICAR has a broad therapeutic potential, since it normalizes the carbohydrate and lipid metabolism and inhibits the proliferation of tumor cells. The synthesis of AICAR in*Bacillus subtilis*cells is controlled by the enzymes of purine biosynthesis; their genes constituting purine operon (*pur*-operon). Reconstruction of purine metabolism in*B. subtilis*was performed to achieve overproduction of AICAR. For this purpose, the gene*purH*, which encodes formyltransferase/IMP-cyclohydrolase, an enzyme that controls the conversion of AICAR to IMP, was removed from the*B. subtilis*genome, ensuring the accumulation of AICAR. An insertion inactivating the gene*purR*that encodes the negative transcriptional regulator of the purine biosynthesis operon was introduced into the*B.subtilis*chromosome in order to boost the production of AICAR; the transcription attenuator located in the leader sequence of*pur*-operon was deleted. Furthermore, the expression integrative vector carrying a strong promoter of the*rpsF*gene encoding the ribosomal protein S6 was designed. The heterologous*Escherichia coli*gene*purF*encoding the first enzyme of the biosynthesis of purines with impaired allosteric regulation, as well as the modified*E.coli*gene*prs*responsible for the synthesis of the precursor of purines — phosphoribosyl pyrophosphate (PRPP) — was cloned into this vector under the control of the*rpsF*gene promoter. The modified*purF*and*prs*genes were inserted into the chromosome of the*B. subtilis*strain.*B. subtilis*strain obtained by these genetic manipulations accumulates 11–13 g/L of AICAR in the culture fluid.

## INTRODUCTION 

**Fig. 1:**
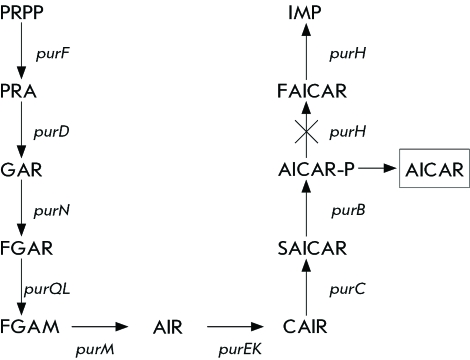
De novo purine nucleotide biosynthesis in *B. subtilis* . Representative enzymatic steps of de novo purine biosynthesis are shown by the corresponding gene designations. Abbreviations: PRPP - 5’-phosphoribosyl-1-pyrophosphate, PRA-5’-phosphoribosylamine, GAR - 5’-phosphoribosylglycineamide, FGAR - 5’-phosphoribosyl-N-formylglycineamide, FGAM - 5’- phosphoribosyl-N-formylglycinamidine, AIR - 5’-phosphoribosyl-5-aminoimidazole, CAIR - 5’-phosphoribosyl-4-carboxy-5-aminoimidazole, SAICAR - 5’-phosphoribosyl-4 (N- succinocarboxamide)-5-aminoimidazole, AICAR-P - 5’- phosphoribosyl - 4-carboxamide-5- aminoimidazole, FAICAR - 5-formamidoimidazole-4-carboxamide ribotide, IMP - inosine 5’-monophosphate, AICAR - 5-aminoimidazole-4-carboxamide 1-β-D-ribofuranoside.

Despite the fact that the structural organization of the genes encoding the enzymes of purine nucleotide biosynthesis is quite versatile, the biochemistry of the process is conservative for different organisms: the formation of the purine cycle occurs on the basis of a riboso-5-phosphate (all intermediates are nucleotides) using a monocarbon component (formiate and/or N10-formyltetrahydrofolate) [1]. There is demand for monocarbon compounds at two stages of purine biosynthesis; therefore, the precursors – phosphoribosylglycineamide ribonucleoside (GAR) and 5-aminoimidazole-4-carboxamide ribonucleoside (AICAR-P) can be accumulated if there is a deficiency in these compounds. Among them, AICAR-P occupies a specific place, since its formulation and subsequent cyclization crown the formation of the purine heterocycle that yields inosine monophosphate (IMP) ( *Fig. 1* ). The process of conversion of AICAR-P into IMP in prokaryotic cells is controlled by the gene *purH* , which encodes two domains with the activities of AICAR-P-formyltransferase and IMP cyclohydrolase [2, 3]. Further modifications of IMP yield AMP and GMP.

Despite the fact that the structure of purine heterocycle is incomplete, AICAR-P is a natural analogue of AMP, substituting it in certain enzymatic *in vitro* reactions. The possibility that AMP could be substituted in the reactions of activation of AMP-activated proteinkinase (AMPK) in mammals has been given a significant degree of attention over the past decade. AMPK is the global regulator of the metabolic processes ensuring the energy status of the eukaryotic organism [4, 5]. For * in vivo* activation of AMPK, it is convenient to use a AICAR nucleoside, which can be rapidly phosphorylated in cells, yielding AICAR-P, an analogue of AMP. The emergence of AICAR-P imitates the accumulation of AMP and provokes the rearrangement of energy processes directed towards the overcoming of imaginary energetic stress. Due to their ability to activate AMPK, AICAR-based drugs have a broad therapeutic potential, since they normalize both the carbon [6] and lipid [7] metabolism. AICAR suppresses tumor cell growth by imitating the state of energetic stress [8]. The efficacy of AICAR in the prevention of type 2 diabetes mellitus has been demonstrated [9]. AICAR induces apoptosis; it is efficient upon chronic [10] and acute leukoses [11].

The present work was aimed at obtaining the strain producer of AICAR by directed reconstruction of purine metabolism in *B. subtilis * cells. The choice of *B. subtilis * was conditioned on the fact that genetic control and regulation of purine metabolism in these bacteria have been subjected to an appropriately thorough study. Furthermore, *Bacillus* strains have been in use for a significant length of time as producers of purine nucleosides and nucleotides, such as inosine and inosinic, as well as guanosinic, acids [12, 13].

Purine operon *B. subtilis, purEKBCSQLFMNHD * (hereafter referred to as *pur* -operon), encodes the enzymes of the synthesis of IMP, the most significant intermediate compound upon purine nucleotide biosynthesis ( *Fig. 1* ).

**Fig. 2:**
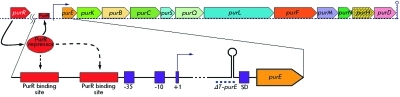
Scheme of structural organization of the *B. subtilis pur-* operonand its regulation. Top: relative location of the 12 linked structural genes that constitute the *pur* -operon and unlinked *purR* gene, encoding a repressor of *pur* -operon. Bottom: the leader region of *pur* -operon, including binding sites for the repressor protein PurR, binding site for RNA polymerase (-35 ; - 10), transcription start (+1), the terminator of transcription (hairpin structure) and the ribosome binding site (SD); the dotted line denotes deletion of the leader region of *pur* -operon ( *∆T-purE* ).

The group consisting of 12 linked genes that form the *pur* -operon is localized at the 55° region on the chromosome of *B. subtilis* ( *Fig. 2* ) [14]. Expression of the *pur* -operon of *B. subtilis * undergoes a double- negative regulation, by the protein-repressor PurR [15] and the transcription attenuator located in the leader sequence of *pur* -operon [16]. It was shown that PRPP acts as a low-molecular-weight effector of the PurR protein [15], whereas guanine serves as a modulator enhancing transcription termination prior to the first structural gene of the operon [17]. Later, it was revealed that a 5’-non-translatable sequence of mRNA has a sensory function with respect to the metabolite – guanine effector, and that it acts as the so-called riboswitch [18, 19], providing early termination of operon transcription [20, 21].

Thus, maximum *pur* -operon gene expression had to be made possible at the first stage of obtainment of the strain producer of AICAR by eliminating the negative regulation of the *pur* -operon under the action of the protein repressor PurR and the transcription attenuator in the leader sequence of the operon. The gene *pur* H encoding formyltransferase/IMP-cyclohydrolase, which participates in the synthesis of AICAR, was then deleted from the genome of the resulting strain. Inactivation of this enzyme is intended to ensure the intracellular accumulation of AICAR ( *Fig. 1* ). At the next stage, the pool of major precursors of *de novo* purine synthesis — PRPP — was to be increased. PRPP is synthesized from riboso-5-phosphate under the control of PRPP synthase encoded by the *prs* gene. This enzyme is susceptible to allosteric regulation with the participation of purine nucleotides. The structural and functional organization of PRPP synthase was studied more thoroughly in *E. coli* bacteria, in which a mutant variant of this enzyme, with eliminated allosteric regulation, was obtained [22]. Taking these data into account, site-directed mutagenesis of the *prs* gene of *E. coli * aimed at obtaining a mutant enzyme that would not be susceptible to retroinhibition by purine nucleotides was preformed with the purpose of its subsequent transfer into *B. subtilis * cells. .

An additional impediment in the effort to boost AICAR production is the allostreric regulation of the first enzyme of purine biosynthesis — glutamine–PRPP aminotransferase encoded by the *purF* gene [23]. It is well known that glutamine–PRPP aminotransferase from *E. coli* , as opposed to the enzyme from *B. subtilis* , is not susceptible to inactivation in the steady-state stage of bacterial growth [24]. Moreover, the mutant variant of this enzyme, which is resistant to inhibition by purine nucleotides, has been described for *E. coli* [25]. Therefore, in our case the decision was made to use the glutamine–PRPP aminotransferase from *E. coli* modified by site-directed mutagenesis with the aim of subsequently transferring it into *B. subtilis * cells. An integrative expression vector based on plasmid pDG268 was constructed comprising a strong promoter of the rpsF gene, which encodes the ribosomal protein S6, in order to ensure the optimal expression of the modified *prs* and *purF* genes of *E. coli* in *B. subtilis * cells. The final stage of the process comprised the integration of the resulting vector, containing clones of the *prs* and *purF* genes under the control of the *rpsF* gene promoter, into the chromosome of the AICAR-producing strain *B. subtilis.*

## experimental 

**Bacterial strains and plasmids**

The bacterial strains and plasmids used in this study are listed in *Table 1* . The *B. subtilis* strain AM732 was obtained via the transformation of the Mu8u5u6 strain of chromosomal DNA isolated from the *B. subtilis * strain 168 with the selection of Pur ^+ ^ transformants. Strain AM743 was obtained by the transformation of strain АМ732 of chromosomal DNA isolated from *B. subtilis * strain LCC28 with the selection of transformants Neo ^R^ in a neomycine-containing medium.

**Media and culturing conditions**

A LB medium [31], or the standard-minimal Spizizen’s medium, with the required additives [26] was used as the nutrient medium to culture bacteria. Aminoacids were added in an amount of 50 µg/mL. Glucose (0.4%) was used as a source of carbon. The following antibiotics were added into the medium when necessary: ampicillin (Amp) – 100 µg/mL; chloramphenicol (Cm) – 10 µg/mL; erythromycin (Em) – 1 mg/mL; and neomycin (Neo) – 5 µg/mL. Methionine and leucine were added at a concentration of 50 µg/mL. Hypoxantine, adenine, or guanine (20 µg/mL) was used as a purine source for purine auxotrophs. The DNA of *B. subtilis * was isolated according to the Saito–Miura procedure [32]; the transformation experiments were carried out in accordance with Anagnostopoulos and Spizizen’s work [26].

**Manipulations with plasmid DNA**

The isolation of plasmid DNA, cloning, transformation into *E. coli* cells, and analysis of recombinant plasmids were performed using the standard methods [31].

**Enzyme preparations**

Restriction endonucleases, T4-DNA-ligase, and thermostable Taq DNA polymerase were purchased from Fermentas International Inc. 

**Polymerase chain reaction**

The PCR was performed on a MyCycler thermal cycler (Bio-Rad Laboratories). The temperature mode was selected with due consideration of the length of the amplified fragment, as well as the length and composition of the primers used. The isolation and purification of the PCR products was carried out using a Silica Bead DNA Gel Extraction Kit (Fermentas International Inc.). 

**Site-directed mutagenesis**

**Table 1:** Bacteria and plasmids used in the present study

Strain or plasmid	Description or genotype	Source or reference
*Bacillus subtilis*		
168	*trpC*	[26]
Mu8u5u6	*leu met purF*	[27]
LCC28	*purR::neo*	[28]
AM747	*trpC purH::(pMutin2purH’-lacZ) ΔT-purE*	[21]
АМ732	*leu met*	This study
AM743	*leu met purR::neo*	- “ -
АМ764	*leu met purR::neo ΔT-purE*	- “ -
АМ778	*leu met purR::neo ΔT-purE ΔpurH*	- “ -
АМ793	*leu met ΔpurH*	- “ -
АМ811	* leu met purR::neo ΔT-purE ΔpurH amyE::[P_rpsF_-prs_E_] *	- “ -
АМ813	* leu met purR::neo ΔT-purE ΔpurH amyE::[P_rpsF_-purF_E_] *	- “ -
АМ815	* leu met purR::neo ΔT-purE ΔT ΔpurH amyE::[P_rpsF_-prs_E_-purF_E_] *	- “ -
*E.coli*		
TG1	* thi supE hsd∆5 ∆(lac-proAB)/F^’^tra ∆36 proAB^(+)^ lacI^(q)^ lacZ ∆M15 *	VKPM
MG1655	prototroph	[29]
Plasmids:		
pDG268	Ap^r^(*E.coli*) Cm^r^(*B.subtilis*)	[18]
pNZT1	Em^r^	[30]
pLE1	as pDG268, but contains a promoter of * rpsF_E_*gene	This study
pLE2	as pLE1, but contains a* prs_E_*gene	- “ -
pLE3	as pLE1, but contains a* purF_E_*gene**	- “ -
pLE4	as pLE1, but contains a*prsE*и* purF_E_*genes	- “ -

Ap ^r^ – ampicillin resistance, Em ^r^ erythromycin resistance, Cm ^r^ – chloramphenicol resistance.

VKPM Russian National Collection of Industrial Microorganisms

Site-directed mutagenesis was performed using specific oligonucleotide primers. The composition and characteristics of the primers are listed in *Table 2* . The presence of corresponding mutations was verified by sequencing according to Sanger [33].

**Fermentation conditions**

The ability of the strains obtained during the study to accumulate AICAR in culture liquid (CL) was assessed. The strain inoculum was cultivated at 37 ^o^ C for 18 h on a LB broth. Then, 0.5 mL of the culture was added to each 20 x 200 mm vial with 4.5 mL of the fermentation medium and cultured at 37 ^o^ C for 72 h on a rotary shaker. The fermentation medium had the following composition (%): soy flour – 3; nutrient yeast – 1; corn extract – 5; (NH _4_ ) _2_ HPO _4_ – 0.6; carbamide – 0.4; and sugar – 15, pH 7.0.

**Determination of AICAR concentration in the culture liquid**

The CL obtained during fermentation was centrifuged to remove the cells; the AICAR concentration was then determined in the supernatant on Sorbfil plates (OOO Lenchrom, Saint Petersburg, Russia) by quantitative thin-layer chromatography. The composition of the eluting system designed was as follows: chloroform–methanol–water–25% aqueous solution of ammonia at a volume ratio of 5 : 3 : 1. Quantitative HPLC on a chromatograph (ALLIANCE, Separations Module Waters 2695, Photodiode Array detector Waters 2996) was employed as an alternative method. 

## RESULTS AND DISCUSSION 

**Enhancement of the expression of the**

*B. subtilis pur* **-operon**

In work [21] devoted to the study of *B. subtilis* *pur* -operon expression, it was demonstrated that an almost 20-fold enhancement of the expression of the *lacZ* reporter gene integrated into the *purH* gene, as compared with the expression in the wild-type strain when the *purR* gene that encodes the repressor protein of the operon is damaged, is achieved. After deletion of 94 n.p. of the wild-type strain, which captures the Rho-independent transcription attenuator ( *ΔT-purE)* located in the leader region of the *pur* -operon ( *Fig. 2* ), the expression of the *lacZ * reporter gene increased approximately by a factor of 10. However, when combining both mutations, a pronounced synergic effect was observed; *lacZ* gene expression increased by a factor of more than 200. Taking these data into account, the experiments on the inactivation of the *purR * gene and deletion of the transcription attenuator ( *ΔT-purE) * were performed at the first stage of construction of the AICAR-producing strain.

**Table 2:** Primers used in this study

Name	Gene	Sequence*	Coordinates **	
			5’	3’
N1	* purN_B_*	cccccgcgggcggaacgattccacat (SacII)	+135	+154
N2	* purN_B_*	cgcctgcagttcttttacgaaaggaacga (PstI)	+652	+630
D1	* purD_B_*	cgcctgcagcttcaaacattaaggggatgaaaa(PstI)	-28	-5
D2	* purD_B_*	cgcggtacctttttcctgcacatatgcc (KpnI)	+410	+389
F1	* purF_E_*	cgcatcgataggaggtgcaaacagatgtgcggtattgtcggtatc (ClaI)	+1	+22
F2	* purF_E_*	cgcgctcagcgaaggcatcatcct (EspI)	+1530	+1511
F3	* purF_E_*	gggcttcgttCaaaaccgctat	+968	+991
F4	* purF_E_*	atagcggttttGaacgaagccc	+991	+968
F5	* purF_E_*	ggtattgatatgTGgagcgccacgg	+1216	+1242
F6	* purF_E_*	ccgtggcgctcCAcatatcaatacc	+1242	+1216
P1	* prs_E_*	cgcggatccaaggaggttcttctcAtgcctgatatga (BamHI)	-21	+3
P2	* prs_E_*	cccatcgatgccgggttcgattagtgttcga (ClaI)	+949	+928
P3	* prs_E_*	ctgacagtggCtctgcacgctg	+366	+377
P4	* prs_E_*	agcgtgcagaGccactgtcagc	+377	+366
R1	* rpsF_B_*	cgcgaattcttgcgggcggcggtat (EcoRI)	-223	-205
R2	* rpsF_B_*	cgcggatccataatgggcaaggagcaat (BamHI)	-31	-51

*The sequence of primers is given in the orientation 5’-3 ‘. Uppercase bold letters indicate the nucleotide substitutions introduced in primers for site-directed mutagenesis. Recognition sites are underlined. Restriction enzymes are shown in parentheses.

**The coordinates of the 5’-and 3’-ends of the primers are relative to the start of translation of the corresponding genes. *B. subtilis* genes are marked with the symbol _(B)_ ; and *E. coli* - with the symbol _ (E)_ .

The AM732-Pur ^+ ^ strain, a derivative of the earlier characterized Mu8u5u6 strain [21] ( *Table 1* ), was used as starting material. In order to transfer the *purR* :: *neo * insertion, which completely inactivates the synthesis of the PurR repressor protein, into the genome of the АМ732 strain, this strain was transformed by chromosomal DNA isolated from the LCC28 ( *purR* :: *neo) * strain, with the selection of recombinants that were resistant to neomycin (Neo ^R^ ). As a result, the АМ743 *purR* :: *neo* strain was selected for use in the subsequent work ( *Table 1* ). The deletion *ΔT-purE * in the genome of the АМ743 *purR* :: *neo * strain was obtained through the following scheme. First, the long-stretched deletion *ΔL-E* was obtained, completely overlapping the leader region of the *pur* -operon and partially overlapping the first structural gene *pur* E, which resulted in the emergence of auxotrophicity with respect to purines. How to achieve this deletion was thoroughly described in [21]. The deletion of the transcription attenuator *ΔT-purE* was transferred into the AM743 *purR* :: *neo ΔL-E * strain by transforming DNA that was isolated from the AM747 strain ( *Table 1* ); Pur ^+ ^ transformants were selected on the purine-free Spizizen’s minimal medium. As a result, the АМ764 strain was obtained. It contained the *purR* :: *neo * insertion and deletion of the transcription attenuator * ΔT-purE * in the genome.

**Obtaining the deletion of the**

*purH* **gene in the** *B. subtilis* **chromosome**

**Fig. 3:**
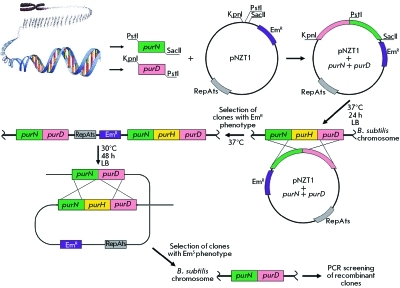
A schematic representation of isolation of the *purH* gene deletion using the method described in [30].

As follows from the schematic representation of purine biosynthesis ( *Fig. 1* ), for intracellular accumulation of AICAR, it is necessary to inactivate formyltransferase/IMP-cyclohydrolase encoded by the *pur* H gene. The PurH gene deletion in the *B. subtilis * chromosome was achieved using the method described in [30] based on using a specially constructed pNZT1plasmid, a derivative of the integrative vector pKS1 [34]. The pNZT1 plasmid contains the erythromycine (Em ^R^ ) resistance marker and a polylinker with multiple restriction sites for cloning the target fragments of chromosomal DNA. Of paramount importance is the fact that its replication is temperature-sensitive: at 30 ^о^ С, the plasmid exists in an autonomous state. However, at 37 ^о^ С the replication is blocked. As a result, if the plasmid has a chromosome fragment cloned into its structure, it is capable of integration into the chromosome by homologous recombination ( *Fig. 3* ). Since plasmid integration into the chromosome results from the single recombination act in the homology region between the cloned fragment and the chromosome, the integration site of the plasmid into the chromosome turns out to be flanked by homologous duplicated sequences. Culturing of these bacteria at 30 ^о^ С (permissive, for pNZT1 plasmid replication) may result in its removal from the chromosome with capture of a copy of the flanked chromosome sequences, which allows for the replacement of the wild allele of any chromosome gene by the mutant gene cloned in the pNZT1 plasmid ( *Fig. 3* ).

This feature of the pNZT1 plasmid was used for the deletion of the *purH * gene. With this aim in mind, the PCR amplification of the chromosome fragments that flanked the *purH* gene was performed. A distal region (517 np) of the *purN* gene adjacent to the 5’-terminus of the *pur* H gene was amplified using N1 and N2 primers, followed by cloning into pNZT1 plasmid at restrictases SacII and PstI digestion sites. The proximal region (442 np) of the *pur* D gene adjacent to the 3’-terminus of the *pur* H gene was amplified at the next stage using D1 and D2 primers ( *Table 2* ), followed by cloning at restrictases PstI and KpnI digestion sites into the pNZT1 plasmid that was obtained at the previous stage and contained the insertion of the *purN* gene fragment. The *E. coli* strain TG1 was transformed by this plasmid. Em ^R^ transformants were sampled from a medium with erythromycin (300 µg/mL) at 30 ^о^ С. The presence of the pNZT1 plasmid with cloned purN and purD gene fragments was tested in the resulting clones using PCR. The АМ764 * purR* :: *neo ΔT-purE * strain was transformed by pNZT1- *purN-purD * plasmids isolated from the inspected clones. The selection of the Em ^R^ transformants was carried out on a medium containing erythromycin (3 µg/mL). Several Em ^R^ clones were cultured at 37 ^о^ С for a night, seeded into plates containing the LB medium with erythromycin (3 µg/mL) and incubated for 24 h at 37 ^о^ С. Em ^R^ recombinants were formed by integration of the pNZT1- *purN-purD * plasmid into the corresponding chromosome locus, which was attested by PCR amplification of the fragment consisting of 2413 bp using N1 and D2 primers. Several Em ^R ^ clones were seeded into an antibiotic-free liquid LB medium and incubated on a shaker at 30 ^о^ С for 48 h. This was followed by seeding onto plates with an antibiotic-free LB medium and incubation for another 24 h at 30 ^о^ С. The excision of the integrated pNZT1 plasmid from the resulting clones was tested. This was detected by the emergence of erythromycin-sensitive clones. As noted above, plasmid excision can be accompanied by either the retention of the wild-type allele of the *purH * gene in the chromosome, or by substitution of this allele for the *ΔpurH * deletion ( *Fig. 3* ). The integration (transfer) of the *ΔpurH * deletion into the chromosome of the АМ764 * purR* :: *neo ΔT-purE * strain was attested by the production of a PCR fragment (with a size of 2413 np) with the participation of N1 and D2 primers. One of the variants of the АМ764 * purR* :: *neo ΔT-purE * strain comprising the *ΔpurH * deletion and called AM778 was capable of accumulating up to 4–5 g/L of AICAR in CL and grew on the minimal medium only upon addition of hypoxantine, adenine, or guanine, suchwise the presence of the defect *purH* gene in its genome was confirmed. Simultaneously, the control strain АМ793 was constructed: it contained *ΔpurH* , but it carried no *purR* :: *neo * and *ΔT-purE * mutations, which provide derepression of the enzymes that involved in purine biosynthesis.

**Fig. 4:**
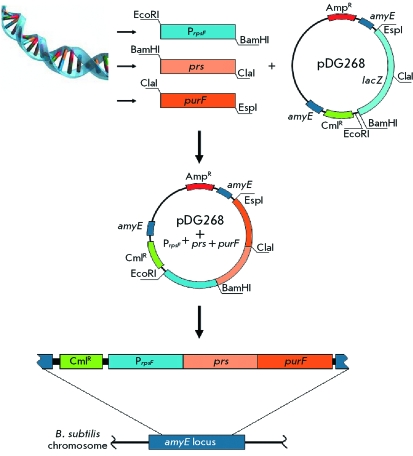
A schematic representation of cloning procedure of *E. coli* *prs* and *purF* genes under the control of the *B. subtilis* *rpsF* gene promoter in the plasmid pDG268, and their integration into the *B. subtilis* chromosome.

It was reasonable to increase the intracellular content of PRPP, the key precursor of purine biosynthesis; in order to enhance the productivity of the strain obtained ( *Fig. 1* ). It is well known that PRPP synthases that are responsible for PRPP synthesis in the cell of both *B. subtilis* [35]and * E. coli * [36] are susceptible to allosteric inhibition by purine nucleotides, including phosphorylated derivatives of AICAR. As previously mentioned in the introduction section, the mutant variant of PRPP synthase of *E. coli, * which is not susceptible to allosteric regulation, has been described [22]. Therefore, the goal was to obtain an analogous mutant enzyme of *E. coli* , to clone it, and to optimize the expression of this enzyme in strain AM778 cells. However, prior to this, the integrative expression vector had to be constructed, which could provide a high level of expression of heterologous *E. coli* genes in *B. subtilis * cells.

**Construction of the integrative expression vector based on pDG268 plasmid**

The pDG268 plasmid was selected as starting material in order to obtain the integrative expression vector. This plasmid can be replicated in *E. coli * (but not * B. subtilis* ) cells. However, when introduced into *B. subtilis * cells, it may integrate into the chromosome locus *amyE * of *B. subtilis* . The pDG268 plasmid comprises a cartridge that includes the polylinker, *lacZ* reporter gene without the intrinsic promoter, and the chloramphenicol (Cm ^R^ ) resistance gene ( *Fig. 4* ). The cartridge is flanked by fragments of the *amyE * gene, which permits integration of the vector with the cloned fragment into the *amyE * locus on the *B. subtilis * chromosome.

**Fig. 5:**
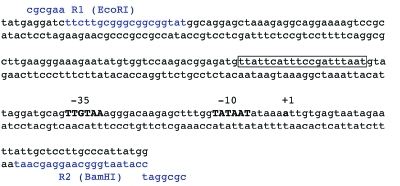
Nucleotide sequence of the * rpsF* gene promoter. -10 and -35 regions shown in bold uppercase. Positions +1 defined as transcription start of *pur* -operon. The UP-element of the promoter is boxed. The nucleotide sequence of the primers used for cloning of the promoter are marked in blue.

The promoter of the *rpsF* gene encoding the ribosomal protein S6 in bacilla was selected as a promoter that is capable of providing a high level of expression of the cloned *E. coli* genes in *B. subtilis * cells. The promoters of ribosomal protein genes have been known to belong to the strongest promoters in *B. subtilis* ; their expression is coordinated with the bacterial growth rate and attains a maximum level at the logarithmic growth stage [37, 38]. The nucleotide sequence of the *rpsF* gene promoter is shown in *Fig. 5.* As follows from *Fig. 5* , the P *rpsF * promoter contains the canonic sequence -70...-50 np, the so-called UP element, which provides a more efficient transcription initiation [39].

With the purpose of cloning the P *rpsF * promoter into the pDG268 plasmid, the DNA fragment containing the promoter region of the *rpsF * gene was amplified by PCR from the *B. subtilis * 168 chromosome using R1 and R2 primers ( *Table 2* ). The resulting PCR fragment was digested with the restriction endonucleases EcoRl and BamHI and cloned into the pDG268 plasmid digested with the same restrictases ( *Fig. 4* ). The *E. coli * TG1 strain was transformed by a ligase mixture. The transformants carrying the insertion of the desired fragment were sampled from an indicator medium containing ampicillin at a concentration of 120 µg/mL and X-gal. Transformant colonies were bright blue, since it was revealed that the *lacZ * reporter gene was controlled by the cloned P *rpsF* promoter. The presence of the insertion was confirmed by PCR using R1 and R2 primers. The resulting plasmid was then integrated into the chromosome *amyE* locus of *B. subtilis * by selecting the chloramphenicol (Cm ^R^ )-resistent transformants. The schematic representation of the integration of the pDG268 vector with the cloned *B. subtilis* *rpsF * gene promoter is provided in *Fig. 4* . The determination of the activity of β-galactosidase in these transformants revealed that the *lacZ* reporter gene expression under control of the P *rpsF * promoter was higher than the expression of this gene when controlled by common promoters, such as the natural *pur* -operon promoter (no data presented), by an order of magnitude. The vector constructed was named pLE1.

**Site-directed mutagenesis of the**

*prs E. coli (prs _E_ )* **gene**

According to the published data, specific mutation in the *prs _E_* gene, resulting in the Asp128 → Ala replacement in PRPP synthase, leads to the removal of retroinhibition of the enzyme by purine nucleotides [22]. In order to achieve the analogous mutation, synthesis of the oligonucleotide primers P3 and P4 ( *Table 2* ) was performed; the primers contain nucleotide replacements (are denoted by uppercase letters) resulting in the formation of a mutant protein with the Asp128 → Ala replacement. At the first stage, PCR fragments were amplified with the participation of two primer pairs: P4–P1 (it flanks the 5’-terminus of the *prs _E _* gene and contains the recognition site for restrictase ClaI). Then, the obtained fragments were joined, and the full-size *prs _E _* gene was amplified using the P1 and P2 primers ( *Table 2* ). It should be emphasized that the nucleotide sequence of the ribosome recognition site (SD) optimized for expression in bacilli is included into the 5’-region of the P1 primer. At the next stage, the P1-P2 PCR fragment containing the mutant *prs _E _* gene was cloned into the earlier obtained pLE1 vector using BamHI and ClaI restrictases. As a result, pLE2 plasmid containing the mutant *prs _E _* gene controlled by the P *rpsF* promoter was obtained. At the final stage, pLE2 plasmid was integrated into the *amyE B. subtilis * chromosome locus according to the earlier described scheme ( *Fig. 4* ). The strain obtained by integration of the pLE2 plasmid was named AM811 ( *Table 1* ).

**Site-directed mutagenesis of the**

*purF E. coli (purF _E_ )* **gene**

The first enzyme of purine biosynthesis, glutamine-PRPP aminotransferase ( *purF* gene), plays the primary role in ensuring the normal functioning of the purine biosynthetic path in *E. coli* and *B. subtilis* . In addition, it is susceptible to allosteric regulation with the participation of nucleotides, which is even more sophisticated in comparison with the PRPP synthase [40]. Therefore, it could be expected that the use of the mutant glutamin-PRPP aminotransferase from *E. coli* that is analogous to that described in [25] will lead to AICAR production. According to [25], Lys326 → Gln and Pro410 → Trp replacements in the protein modify the GMP (A-site) and AMP binding site (C-site), respectively. Combination of these mutations leads to the enzyme being resistant to almost any purine nucleotide. Site-directed mutagenesis of the *purF _E _* gene was performed according to the scheme described in the previous section. The F3 and F4 oligonucleotide primers were synthesized to obtain the Lys326 → Gln replacement; and F4 and F5 primers, to obtain the Lys326 → Gln replacement (in *Table 2* , the corresponding nucleotide replacements are highlighted by uppercase letters and set off in bold). After the PCR amplification of these primers with the flanking primers F1 and F2, followed by the joining of the PCR fragments, the full-size *purF _E_* gene encoding the protein with both amino acid replacements was obtained. As found in the case of the *prs _E_* gene, the SD site optimized for bacilli was introduced into primer F1. The modified *purF _E_* gene was cloned into the pLE1 plasmid under the control of the P *rpsF * promoter at the ClaI and EspI sites. The modified *purF _E_* gene was cloned into the pLE2 plasmid containing the *prs _E_* gene using the same procedure. The corresponding plasmids were named pLE3 and pLE4 ( *Table 1* ). At the final stage, plasmids pLE3 and pLE4 were integrated into the *amyE B. subtilis * chromosome locus according to the scheme described above ( *Fig. 4* ). The strains obtained by integrating plasmids pLE3 and pLE4 were named АМ813 and АМ815, respectively ( *Table 1* ).

**Determination of the ability of strains to accumulate AICAR**

**Fig. 6:**
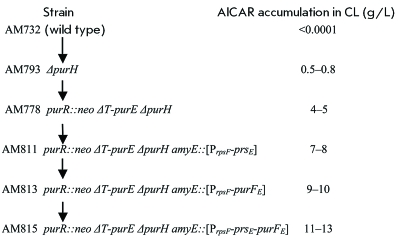
Construction stages of AICAR-producing strains and their productivity.

The fermentation experiments were carried out under the conditions described in the experimental section, in order to estimate the ability of the strains obtained in this study to accumulate AICAR. The results of these experiments are summarized in *Fig. 6* .

As follows from the data given in *Fig. 6* , the initial АМ732 strain accumulates almost no AICAR. Only after the purH gene has been removed from the genome of this strain (AM793 strain) is a negligible (< 1 g/L) accumulation of AICAR in CL observed. The mutations of *purR::neo* and *ΔT-purE * (AM788 strain) ensuring complete derepression of the enzymes of purine biosynthesis result in a considerable accumulation of AICAR; up to 4–5 g/L. A subsequent, almost two-fold enhancement of productivity was observed for the AM811 and AM813 strains expressing one of the mutant desensibilized *E. coli* proteins — PRPP synthase ( *prs* gene) or glutamine-PRPP aminotransferase ( *purF* gene). The maximum accumulation of AICAR (up to 11–13 g/L) was detected for the AM815 strain, which is characterized by the complete derepression of enzymes of purine biosynthesis and simultaneously contains the modified *prs _E _* and *purF _E _* genes controlled by the P *rpsF * promoter.

It is of interest that almost all of the AICAR synthesized in cells is excreted in CL: it was demonstrated in special experiments that the intracellular concentration of AICAR is no higher than 2% of its concentration within the medium. The mechanism of AICAR excretion remains unknown, although data was obtained indicating that the membrane protein encoded by the *pbuE * gene is involved in its export from bacilli cells [41].

The different stages in constructing the AICAR producer are directed towards the stimulation of purine nucleotide biosynthesis; therefore, the AICAR that is detected in CL is formed upon dephosphorylation of the AICAR-P nucleotide synthesized *de novo,* as opposed to being the secondary product of histidine biosynthesis. Since AICAR-P is the natural analogue of AMP, while AICAR is the analogue of adenosine, the increased content of these compounds in producer cells is apparently accompanied by multiple metabolic events (see above); their impact as regards AICAR production is far from clear. In a microorganism, AICAR-P cells serve not only as an intermediate of purine metabolism, but also as a regulatory molecule of all-cell significance. The transformation of AICAR-P into IMP requires the participation of N10-formyltetrahydrofolate; therefore, the increase in the level of AICAR-P in cells may be a warning of monocarbon metabolism disorder, which earlier has made it possible to regard this nucleotide as an alarmone [42]. Regardless of the fact that prokaryotes do not have a target for AICAR-P that would be similar to animal AMPK, the directionality of the physiological action of this AMP analogue is retained in them. In particular, inactivation of the *purH* gene in *Salmonella enterica * and AICAR-P accumulation in cells result in the suppression of the activity of fructose-1,6-bisphosphate phosphatase. As a result, the cells lose their ability for gluconeogenesis and stop growing on glycerol and other gluconeogenic substrates [43]. In cells of prokaryotes and lower eukaryotes (e.g., yeast), a certain amount of AICAR-P is formed as a side product of histidine biosynthesis, which allows additional opportunities for the regulation of purine nucleotide biosynthesis [44]. The numerous regulatory bonds of AICAR-P that remain incompletely studied complicate the construction of producing strains and require further investigation.

## CONCLUSIONS 

The strain-producer of AICAR, a new drug with potentially wide therapeutic applications, was obtained in studies based on *B. subtilis * bacteria. The strategy obtaining the AICAR producing strain is based on the directed reconstruction of purine metabolism in *B. subtilis * cells. At the first stage of the study, an insertion was introduced into the *purR* gene encoding the *pur* -operon repressor protein, and the transcription attenuator was removed from the leader region of the *pur* -operon, ensuring maximum derepression of the enzymes of *de novo* purine biosynthesis. The *purH* gene encoding formyltransferase/IMP-cyclohydrolase was then removed from the bacterial genome. Inactivation of this enzyme disturbs the reaction of AICAR conversion into IMP and results in its accumulation in the cell. At the next stage, the site-directed mutagenesis of *prs* and *purF* *E. coli* genes encoding the key enzymes of the synthesis of purine precursors was carried out in order to obtain mutant variants of these genes that would not be susceptible to retroinhibition by purine nucleotides. Finally, at the last stage, the modified *prs* and *purF* *E. coli * geneswere integrated into the *B. subtilis * chromosome under the control of a strong promoter ensuring a high level of expression of these genes in *B. subtilis * cells. As a result, we obtained a producing strain accumulating 11–13 g/L of AICAR in CL.

To summarize, we would like to note that it was reported recently that AICAR had successfully passed the stage IIa of clinical trials as a antitumor agent [45]. AICAR has a positive effect upon chronic lymphocytic leukemia, multiple myeloma, and mantle cell lymphoma. It should be emphasized that the cost of commercial AICAR substances in catalogues varies from US$100 to 1,000 per gram, which is likely due to the fact that they are prepared by chemical synthesizing. The high cost makes AICAR inaccessible for research and a fortiori for treatment of the metabolic syndrome. The AICAR-producing strain constructed by us may lay the foundations for industrial microbiological production of an affordable AICAR substance that would cost substantially less.
